# Study of Anti-Malarials in Incomplete Lupus Erythematosus (SMILE): study protocol for a randomized controlled trial

**DOI:** 10.1186/s13063-018-3076-7

**Published:** 2018-12-20

**Authors:** Nancy J. Olsen, Judith A. James, Cristina Arriens, Mariko L. Ishimori, Daniel J. Wallace, Diane L. Kamen, Benjamin F. Chong, Duanping Liao, Vernon M. Chinchilli, David R. Karp

**Affiliations:** 1 Division of Rheumatology, Department of Medicine, Penn State MS Hershey Medical Center, 500 University Drive, Hershey, PA 17033 USA; 2 Oklahoma Medical Research Foundation, 825 N.E. 13th Street, Oklahoma City, OK 73104 USA; 3 Cedars-Sinai Medical Center, 8723 W. Alden Drive, Los Angeles, CA 90048 USA; 4 Medical University of South Carolina, 96 Jonathan Lucas St # 912, Charleston, SC 29425 USA; 5 University of Texas Southwestern Medical Center, 5323 Harry Hines Blvd, Dallas, TX 75390 USA; 6 Penn State College of Medicine, 90 Hope Drive, Hershey, PA 17033 USA

**Keywords:** Systemic lupus erythematosus, Randomized clinical trial, Hydroxychloroquine, Incomplete lupus, Undifferentiated connective tissue disease, Prevention

## Abstract

**Background:**

Onset of systemic lupus erythematosus (SLE) is preceded by a preclinical phase characterized by expression of autoantibodies and nonspecific clinical symptoms. Hydroxychloroquine is a treatment for lupus that is widely used based on longstanding experience and a very good safety profile. Existing data suggest that treatment with hydroxychloroquine may postpone the onset of disease. However, prospective studies that prove and quantify the efficacy of hydroxychloroquine in the preclinical phase of lupus have not been done. This study will test the hypothesis that early hydroxychloroquine use can prevent accumulation of clinical abnormalities and modify immune responses that define SLE.

**Methods:**

A randomized, double-blind, placebo-controlled trial of hydroxychloroquine vs placebo will be conducted. Participants will have incomplete lupus erythematosus as defined by the presence of antinuclear antibody (ANA) positivity at a titer of 1:80 or greater, as well as one or two additional criteria from the 2012 Systemic Lupus International Collaborating Clinics (SLICC) classification criteria. The age range will be 15–45 years and the treatment phase will be 96 weeks. The primary endpoint will be the increase in the number of features of SLE defined by the 2012 SLICC classification schema. Secondary outcomes will include the proportion of participants who transition to a classification of SLE as defined by SLICC criteria.

**Discussion:**

A major challenge for improving therapies in patients with SLE is early detection of disease. The ANA test that is widely used to screen for SLE has low specificity and interpretation of its significance is challenging. The Study of Anti-Malarials in Incomplete Lupus Erythematosus (SMILE) trial will provide insights into the appropriate target population for intervention, and will assess whether hydroxychloroquine can slow progression as measured by the accumulation of criteria. Ophthalmologic safety in this population will be assessed. The study will investigate candidate biomarkers that will guide treatment decisions and will accumulate a specimen biobank that will be available to the lupus research community for further in-depth mechanistic studies. This trial is a first step toward testing the feasibility of disease prevention strategies in SLE.

**Trial registration:**

ClinicalTrials.gov, NCT 03030118. Registered on 24 January 2017.

**Electronic supplementary material:**

The online version of this article (10.1186/s13063-018-3076-7) contains supplementary material, which is available to authorized users.

## Background

Systemic lupus erythematosus (SLE) is an autoimmune disease that causes major organ damage and shortens the lifespan. It has a disproportionate impact on young people, especially women. Despite advances in treatment, morbidity and mortality in this largely young population are unacceptably high. This is in part because many patients present at a stage when organ damage is already present. A key to improving outcomes in any chronic disease is early identification and treatment, prior to the onset of irreversible changes [1]. A major challenge to this approach in SLE has been a lack of biomarkers that confidently identify individuals in early disease stages. A positive test for antinuclear antibody (ANA) is generally required to establish an SLE diagnosis, but most individuals with this finding do not progress to SLE [2]. The finding that other autoantibodies more specific for SLE are present years prior to onset of clinical symptoms has suggested it is possible to identify early at-risk patients [3]. A body of work has been developed by us and others in the past decade around the concept that individuals with clinical and laboratory features suggestive of SLE, but who do not fulfill classification criteria for this disease, have incomplete lupus, or ILE, and that this group includes a significant proportion who are at risk for progression to SLE [4, 5]. Blood profiling, including not just autoantibodies but also cytokines and expressed genes, may be useful for identification of those who are at sufficiently high risk to justify therapeutic intervention [6, 7].

Identification of an at-risk population would not be especially useful without an available therapeutic approach. An acceptable drug treatment would be one that offers potential for disease modification while at the same time presenting an acceptable safety profile. An available medication that fits this description is hydroxychloroquine (HCQ). Data from a retrospective analysis of patients who had symptoms suggestive of lupus but who did not fit classification criteria for SLE demonstrated that HCQ could delay full disease onset [8]. Other limited observational studies suggest that autoantibody profiles can be modified by HCQ treatment [9]. Although HCQ is used commonly in clinical practice where it is thought to improve outcomes of ILE patients, no controlled trial data are available to document the clinical, immunologic and adverse effects of HCQ in a population at risk for SLE.

The objectives of the SMILE trial are to determine whether HCQ should be recommended for ILE patients and to provide insights into the appropriate target population. While not part of the Precision Medicine Initiative®, the SMILE trial is consistent with its goals. This trial will be the first step toward testing the feasibility of disease prevention studies in SLE and will accumulate biological samples in a repository that will be available to the lupus research community for further in-depth mechanistic studies.

## Methods

### Study setting and subjects

The study population will consist of patients who have an ANA by immunofluorescence assay on Hep-2 cells of 1:80 or greater and who have one or two additional criteria from the 2012 Systemic Lupus International Collaborating Clinics (SLICC) classification criteria. The age range is 15–45 years. Subjects will be recruited in five US academic medical centers: Penn State MS Hershey Medical Center, Hershey, PA; University of Texas Southwestern Medical Center, Dallas, TX; Oklahoma Medical Research Foundation, Oklahoma City, OK; Cedars-Sinai Medical Center, Los Angeles, CA; and Medical University of South Carolina, Charleston, SC. Appointments, laboratory tests and research samples are free of cost to patients.

### Study design

A double-blind, placebo-controlled design is the optimal approach, due to the variability of ILE and its associated manifestations. Furthermore, randomization permits equalization of variables that are difficult to control at each site, such as sun exposure differences. The design was considered to have equipoise given the current status of ILE treatment, for which many patients do not take regular medications or have close follow-up, while others are given HCQ or other nonimmunosuppressive medications such as nonsteroidal anti-inflammatory drugs (NSAIDs). The risk of placebo is offset by more frequent monitoring and testing of enrolled subjects than would be done in routine clinical practice. Furthermore, any patient who accumulates sufficient criteria to be classified as SLE will be withdrawn to receive standard of care. This study was determined by the Food and Drug Administration (FDA) to be exempt from investigational new drug (IND) status.

Standard protocol items on the list of recommendations for interventional trials (SPIRIT) are provided as a completed checklist (Additional file 1).

### Inclusion and exclusion criteria

Since ANA positivity is critical to the definition of ILE, each enrolled individual will be expected to have had a level of 1:80 or greater recorded as part of clinical care and then will be required to have this level of positivity confirmed by the immunofluorescent assay that is considered the standard, carried out at the Oklahoma Medical Research Foundation (OMRF) CAP/CLIA-certified Clinical Immunology Laboratory. The SLICC criteria were chosen to classify ILE and SLE due to the greater sensitivity compared to the previous American College of Rheumatology (ACR) criteria [10]. Both female and male patients of any race or ethnicity may be enrolled, but ILE is predominantly a female condition so the expectation is that most subjects will be female.

The inclusion criteria are presented in Table 1 and the exclusion criteria are presented in Table 2.

**Table 1:** Study of Anti-Malarials in Incomplete Lupus Erythematosus (SMILE) inclusion criteria

1.	Between 15 and 45 years of age, inclusive, at Visit 1
2.	Antinuclear antibody titer of 1:80 or greater at Visit 1, as determined by immunofluorescence assay in the Oklahoma Medical Research Foundation laboratory
3.	Participants must have at least one (but not three or more) additional clinical or laboratory criterion from the 2012 Systemic Lupus International Collaborating Clinics classification criteria
4.	Written informed consent (and assent when applicable) obtained from subject or subject’s legal representative and ability for subject to comply with the requirements of the study, including willingness to take hydroxychloroquine

**Table 2:** Study of Anti-Malarials in Incomplete Lupus Erythematosus (SMILE) exclusion criteria

1.	Subject meets the 2012 Systemic Lupus International Collaborating Clinics classification criteria for systemic lupus erythematosus at Visit 1 (i.e., ANA plus three other criteria or ANA plus biopsy-proven lupus nephritis)
2.	Subject has been diagnosed with another autoimmune disorder, other than autoimmune thyroid conditions
3.	Subject has fibromyalgia, based on clinical history and examination
4.	Subject has previously been or is currently being treated with oral antimalarial agents including hydroxychloroquine, chloroquine or quinacrine
5.	Subject is currently or has been treated with immunosuppressive, immune-modifying or cytotoxic medications as detailed in the protocol
6.	Use of any investigational agent within the preceding 12 months
7.	History of primary immunodeficiency
8.	Active bacterial, viral, fungal or opportunistic infection
9.	Evidence of infection with human immunodeficiency virus, hepatitis B or hepatitis C
10.	Concomitant malignancy or history of malignancy with the exception of adequately treated basal or squamous cell carcinoma of the skin, or carcinoma in situ of the cervix
11.	Subject has significant findings on ophthalmological examination that, in the opinion of the examining ophthalmologist, prevent safe use of hydroxychloroquine.
12.	Subject has other contraindications to treatment with hydroxychloroquine, including preexisting ocular disease, hepatic impairment, psoriasis, porphyria, or allergy to the drug or class
13.	Comorbidities requiring systemic corticosteroid therapy > 10 mg of prednisone per day, or equivalent, or a change in corticosteroid dose within the 3 months prior to Visit 1
14.	Pregnant, breastfeeding or unwilling to practice birth control during participation in the study
15.	Presence of a condition or abnormality that, in the opinion of the investigator, would compromise the safety of the patient or the quality of the data
16.	Inability to comply with the study visit schedule and procedures

*ANA* antinuclear antibody

### Study procedures

**Fig. 1:**
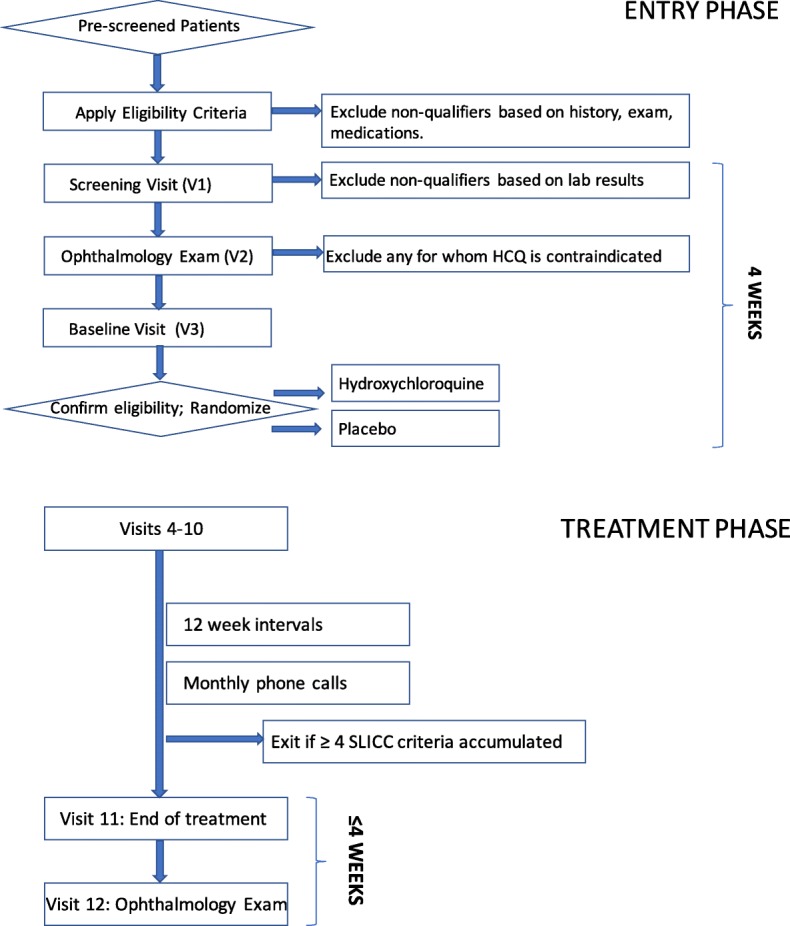
Schematic of initial or entry phase of the SMILE trial from prescreening, through screening and randomization, followed by treatment phase, through the final ophthalmology visit. SPIRIT Standard Protocol Items: Recommendations for Interventional Trials

**Fig. 2:**
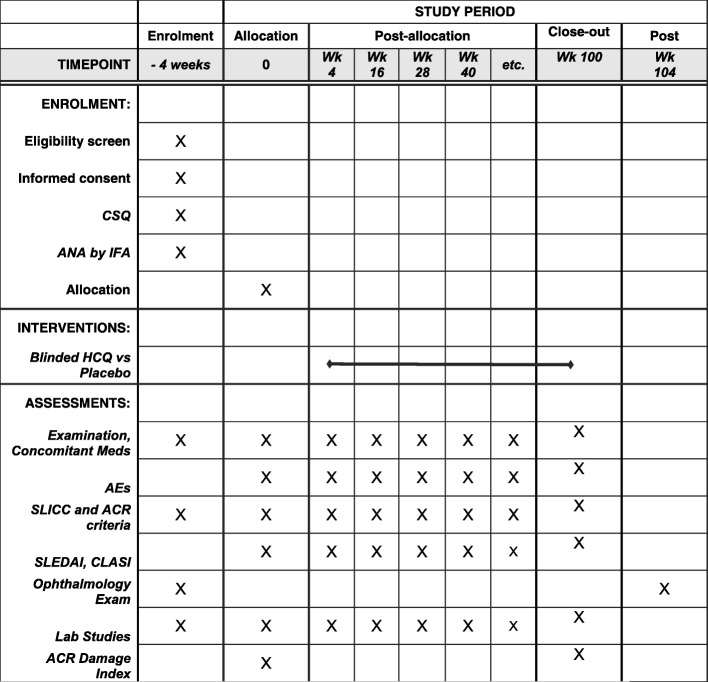
SPIRIT figure of participant timelines. ANA antinuclear antibody, ACR American College of Rheumatology, AE adverse event, CLASI Cutaneous Lupus Erythematosus Disease Area and Severity Index, CSQ Connective Tissue Disease Screening Questionnaire, HCQ hydroxychloroquine, IFA immunofluorescence assay, SLEDAI Systemic Lupus Erythematosus Disease Activity Index, SLICC Systemic Lupus International Collaborating Clinics, SPIRIT Standard Protocol Items: Recommendations for Interventional Trials, Wk week

Prescreened patients, who are in the specified age range, have ANA positivity and have at least one other SLICC criterion point, will be scheduled for a screening visit (Figs. 1 and 2). After obtaining written, informed consent, the subject will complete the Connective Tissue Disease Screening Questionnaire (CSQ) [11]. A medical history, a family history and a physical examination will be completed. Concomitant medications will be recorded. Laboratory testing will be done, including complete blood count with differential, blood chemistry, urinalysis, urine protein/creatinine ratio, pregnancy test, Coombs’ test, anti-phospholipid antibody panel and Complements 3 and 4. A glucose-6-phosphate dehydrogenase level will be obtained to confirm eligibility to receive HCQ. A serum sample will be sent to the OMRF laboratory for measurement of ANA by IFA. Patients who pass screening will undergo an ophthalmologic examination with a dedicated ophthalmologist at each site, to include a dilated retinal examination, a Humphrey 10–2 visual field test and spectral domain optical coherence tomography. Individuals who pass the screening laboratory testing and the ophthalmologic examination, indicating safety for HCQ, will be scheduled for a baseline visit, to take place within 4 weeks of the screening visit.

At the baseline visit (randomization visit), the patient will first complete the self-report Patient-Reported Outcomes Measurement Information System (PROMIS) 29 Adult or Pediatric Profile, supplemented with additional questions from the PROMIS Fatigue item bank, and a Patient Global Visual Analogue Scale. A physical examination and review of concomitant medications and events will be carried out by the investigator. The following items will be completed: two sets of classification criteria for lupus, the SLICC 2012 and the ACR 1997 versions; the Modified SLEDAI-2 K disease activity instrument for SLE that includes the provider’s global assessment (PGA) and SLE Flare Index; the Cutaneous Lupus Disease Area and Severity Index (CLASI) [12]; and the SLICC/ACR Damage Index [13]. A physical examination and review of medications will be carried out and a final check of eligibility will be performed. Blood and urine samples are sent to local and central (OMRF) laboratories. All specimens collected from patients are barcoded according to the Subject ID obtained from the Interactive Web-Based Randomization Service.

Participants will then be randomly assigned to receive HCQ or matching placebo in a 1:1 allocation using a centralized system in REDCap that is stratified by treatment site and number of baseline SLICC classification criteria, either ANA plus one criterion or ANA plus two criteria. To enhance the balance of HCQ and placebo assignments and to reduce the likelihood of unintentional unmasking, a varying permutation block randomization scheme is implemented. Medication is dispensed with instructions to take 2 capsules once a day for participants weighing > 40 kg and one capsule once a day for participants ≤ 40 kg.

Although HCQ has an excellent safety record for use during pregnancy in SLE and similar conditions, all female subjects must use a medically acceptable form of contraception during this study. Pregnancy itself may have effects on the immune system and immune function that cannot be accounted for. A urine pregnancy test will be obtained from female subjects who are of childbearing age prior to their participation in the study, and at weeks 52 and 100.

The study participants will be followed for a total of 104 weeks, including 96 weeks on treatment (Fig. 1). After randomization at Visit 3, Visits 4–11 will occur at 12-week intervals ± 7 days. Each study visit will include medication and adverse event review, physical examination, completion of the SLEDAI, SLICC criteria and ACR criteria. Blood and urine samples for local and central laboratories will be collected, medication compliance determined by pill count and a new supply of capsules dispensed. At approximately 1-month intervals, study personnel will call each subject to reinforce compliance with study procedures. Any potential adverse events or significant changes in the subject’s medical condition will prompt an unscheduled study visit. Unscheduled visits may be performed to document adverse events, worsening of the subject’s medical condition or withdrawal from the study. If the subject has developed changes to suggest a diagnosis of lupus, even if classification criteria are not achieved, then that individual can be withdrawn from the study at the discretion of the site investigator. Within 4 weeks of completion of the treatment period, participants will undergo another ophthalmological examination (Visit 12, week 104).

### Blinding

Participants and the clinical study staff will be blinded as to which treatment is administered. Study pharmacists at each site will not be blinded to group assignment, although the identity of each group, designated A or B, will not be revealed to them. Randomization is done with varying permuted block sizes at each site to minimize inadvertent unblinding of treatment group assignment. All other personnel, including study monitors and statisticians, will remain blinded to study treatment until all subjects have completed the trial and the database is locked. Every effort will be undertaken to maintain the blinding of study treatment. If a participant has a medical emergency, the site principal investigator may break the blind by contacting their local investigational pharmacy. This should be done only if it will affect the participant’s immediate medical care and after discussion with the Medical Monitor and Sponsor. Information for unblinding at each site will be kept in a printed document that will be stored in a secure location and will be used by the site investigational drug pharmacist. The site investigator is responsible for obtaining from the Sponsor the permission to unblind a patient before informing the site pharmacist to carry out the unblinding procedure.

### Study groups

#### Intervention

HCQ tablets will be purchased from commercial generic pharmaceutical vendors that have been approved by the US Food and Drug Administration for sale in the United States. University of Iowa Pharmaceuticals (UIP) will overencapsulate 200 mg HCQ tablets, filling the capsules with microcrystalline cellulose.

### Placebo

Placebo capsules consisting of microcrystalline cellulose and identical in size and shape to the hydroxychloroquine capsules will be manufactured by UIP.

The study drug, either HCQ or matching placebo, will be packaged by UIP in bottles containing 100 capsules each. The bottles will be packaged into kits by the University of Rochester Clinical Materials Services Unit*.*

#### Data collection and management

The data will be entered into a validated REDCap database maintained at the Penn State College of Medicine Data Management Unit. REDCap (Research Electronic Data Capture) is a secure, web-based application designed to support data capture for research studies, providing: an intuitive interface for validated data entry; audit trails for tracking data manipulation and export procedures; automated export procedures for seamless data downloads to common statistical packages; and procedures for importing data from external sources [14]. The Data Management Unit, functioning as the Data Coordinating Center (DCC), will be responsible for data processing, in accordance with procedural documentation. Database lock will occur once quality assurance procedures have been completed. All procedures for the handling and analysis of data use good computing practices meeting FDA guidelines for the handling and analysis of data for clinical trials. Protocol compliance will be monitored by the staff of the DCC in the Department of Public Health Sciences, Penn State College of Medicine. Personnel from the DCC will routinely check each electronic case report form (eCRF) for validity and consistency and immediately ask research coordinators to resolve any discrepancies. This will include reviewing subject eligibility at entry and throughout the trial. Personnel from the DCC will also make two in-person trips to each site to review source documents, investigational drug control and compliance with local regulatory authorities. Any noncompliance by site investigators such as failure to resolve eCRF queries, failure to follow protocol procedures or recruitment of ineligible subjects will be reported immediately to the Sponsor.

The DCC maintains a website where study documents, training videos and the manual of operating procedures are maintained and updated. Meeting announcements and minutes are also posted. Access to the website is by invitation and is password protected.

The sponsoring institute of the National Institutes of Health (NIH), the National Institute of Arthritis and Musculoskeletal and Skin Diseases (NIAMS), has appointed an independent Safety Officer and Data Safety Monitoring Board (DSMB) for this study. Progress reports, including participant recruitment, retention/attrition and adverse events (AEs), will be provided to the Safety Officer on a quarterly basis, and semi-annually to the DSMB. The semi-annual DSMB report will include a list and summary of AEs. In addition, the DSMB report will address: whether AE rates are consistent with prestudy assumptions; reasons for dropout from the study; whether all participants met the entry criteria; whether continuation of the study is justified; and conditions whereby the study might be terminated prematurely. The DSMB report will be sent to the Safety Officer who will forward it to the DSMB and NIAMS. Following review of the report, the DSMB will make recommendations to the Principal Investigator and NIAMS for the continued conduct of the study.

### Evaluation of outcomes

The primary outcome is the increase in the number of clinical and/or laboratory features of SLE defined by the 2012 SLICC classification criteria between week 4 and week 100. Secondary outcomes include the proportion of participants who transition to a classification of SLE according to the 2012 SLICC criteria and the 1997 ACR criteria. Other secondary outcomes are changes in the SLE Disease Activity Index (SLEDAI)-2 K and Cutaneous Lupus Erythematosus Disease Area and Severity Index (CLASI) scores and the frequency of clinically relevant autoantibodies (anti-dsDNA, anti-Sm, anti-Ro, anti-La) or other laboratory findings (C3, C4). Abnormalities in the ophthalmologic assessments will be determined. Exploratory outcomes include changes in panels of autoantibodies, cytokines and other soluble mediators.

### Sample size calculation

The target sample size to be analyzed for this trial is 240 patients (120 randomized to each of the placebo and HCQ groups). For the primary outcome variable of the SLICC classification criteria count, it is anticipated that the probability of the SLICC classification criteria count increasing by at least one criterion during the minimum 96-week follow-up period is 0.4 for a placebo patient and 0.2 for an HCQ patient. There is 90% statistical power to reject the null hypothesis with a two-sided 0.05 significance level if 192 subjects can be analyzed, allowing for 20% dropout over 96 weeks. Up to 300 subjects may need to be consented to account for screen failure prior to randomization.

### Statistical analysis

The primary outcome variable in this trial is the SLICC classification criteria count, measured every 12 weeks over a 96-week period. Ordinal logistic regression analysis with random effects will be applied to compare the estimated slopes for the HCQ and placebo groups with respect to the SLICC classification criteria count over the 96-week follow-up period. The primary null hypothesis is that the HCQ slope equals the placebo slope, and its corresponding alternative hypothesis is that the HCQ slope does not equal the placebo slope. Thus, a two-sided test with a 0.05 significance level will be applied. The statistical model will account for the ordinal and nondecreasing properties of the SLICC classification criteria count during the 96-week follow-up period, as well as account for the repeated measurements on each patient. This statistical model will form the basis of the primary statistical analysis of the SLICC classification criteria count, as well as all secondary and subgroup analyses with the SLICC classification criteria count.

The important secondary outcomes variables in this 96-week trial are: (1) the time to progression from ILE to clinical SLE; (2) disease activity as measured by the SLEDAI and CLASI scores and patient-reported outcomes as measured by PROMIS questionnaires at each visit; (3) concentrations of at least 50 soluble mediators and the number of positive autoantibodies (out of approximately 100 autoantibodies) detected; and (4) the incidences of HCQ-related ophthalmologic toxicity. Outcome variables in items (2) and (3) are measured at the baseline and at each follow-up visit, and can be treated as continuous variables—except for the proportion of positive antibodies, which will be regarded as having a binomial distribution. A linear mixed-effects model will be applied to compare the estimated slopes for the HCQ and placebo groups with respect to the concentration of each soluble mediator. A nonlinear mixed-effects model with a binomial distribution will be used for the cumulative number of positive autoantibodies over the 96-week follow-up period. A binomial regression analysis with a logit link function and random effects will be applied to compare estimated slopes for the two treatment groups. Because a longitudinal data analysis will be applied to each mediator, Hochberg’s step-down procedure will be imposed to manage the family-wise significance level of the set of analyses. Safety variables in item (4) are measured twice, prior to baseline and at the end of the treatment period, as binary variables. No interim analyses are planned.

Because the time to progression from ILE to clinical SLE will be assessed at each follow-up visit, it is interval censored. The analysis will use a discrete time-to-event hazard model [15]. In effect, this model reduces to a logistic regression model with a complementary log–log link function. The concentrations of soluble mediators are measured at each follow-up visit, so a linear mixed-effects model will be used to compare the estimated slopes for the HCQ and placebo groups with respect to each mediator over the 96-week follow-up period. Safety and tolerability data will be summarized by treatment group, with descriptive statistics, and tested for association with treatment by chi-square test or Fisher’s exact test, as appropriate.

Adverse events (AEs) will be tabulated by treatment group and will include the number of patients for whom the event occurred, the rate of occurrence, the severity and the relationship to the study drug.

### Ethical considerations

It might be argued that HCQ is already commonly used in clinical practice for conditions like ILE, and that therefore this precludes the need for a clinical trial. However, the documentation of both efficacy and safety of HCQ for ILE remains unknown in the absence of available randomized controlled trial data. It is possible that ILE patients are actually overtreated with HCQ, with low but unacceptable rates of eye toxicity. Therefore, the placebo-controlled design has equipoise.

The study will be conducted according to the Declaration of Helsinki, Protection of Human Volunteers (21 CFR 50), Institutional Review Boards (21 CFR 56) and Obligations of Clinical Investigators (21 CFR 312). To maintain confidentiality, all laboratory specimens, evaluation forms, reports and other records will be identified by a coded number and initials only. All study records will be kept in a locked file cabinet. Clinical information will not be released without the written permission of the subject, except as necessary for monitoring by the FDA or regulatory agencies. Written, informed consent will be obtained by a designated member of the study team from each subject prior to entering the subject into the trial. Information will be given in both oral and written form and subjects (or their legal representatives) will have ample opportunity to have all questions answered. If appropriate and required by the local institutional review board (IRB) or ethics committee, assent from the subject will also be obtained. If a subject is unable to sign the informed consent form (ICF) and the Health Insurance Portability and Accountability Act (HIPAA) authorization, a legal representative may sign for the subject. A copy of the signed consent (and assent) form will be given to the subject or the subject’s legal representative, and the original will be maintained with the subject’s records. This study was approved by institutional review boards at each of the five study sites.

## Discussion

A challenge to improving treatment for SLE is the capability to reliably identify individuals who are in the early disease stages and who are at risk for organ-damaging disease. The usual screening starts by measuring ANA. Since ANA positivity is essentially required for SLE diagnosis, it is a useful filter that permits enrichment for a segment of the population in which SLE is likely to occur. However, the problem with ANA testing is the high prevalence of positivity, at least at low levels, in the general population, which may approach 25% [16, 17]. Interpretation of ANA positivity in persons with vague or inconsistent symptoms is not straightforward, and identification of the small number of ANA-positive individuals who are developing SLE is challenging.

The presence of suggestive clinical features likely adds to the SLE risk. This is where the ILE model is especially useful. As first described by others [18], these patients exhibit some features of lupus but do not satisfy sufficient criteria for classification as SLE [2, 6, 16, 19, 20]. A significant subset of ILE patients is likely to progress to SLE.

Given the relative safety and tolerability of HCQ, many physicians prescribe this medication for patients who present with evidence of autoimmunity (i.e., an ANA) and some evidence of immune pathology such as a rash or joint inflammation, or even just constitutional symptoms of arthralgia and fatigue. However, the efficacy of HCQ in reducing either the objective signs or patient-reported symptoms has not been tested prospectively in this population. While HCQ is usually well tolerated, it has known side effects and its use requires regular safety monitoring. This adds risk and cost to the management of patients with a drug that has not undergone specific testing for this indication. While some recommendations suggest annual ophthalmologic evaluation, risks are related to the dose and duration of the therapy, both of which are low in this trial, and therefore baseline and endpoint examinations were judged to be safe.

The availability and use of an unproven, but relatively safe, drug for people with ILE as well as the reluctance of some potential participants to take medication before they develop classifiable SLE are major barriers to recruitment. This protocol was selected for assistance from the National Center for Advancing Translational Sciences-supported Trial Innovation Network Recruitment Innovation Center (RIC). Culturally appropriate recruiting materials were created in English and Spanish. Letters for local physicians were developed by the RIC. Screening tools that provide study personnel with lists of potentially eligible subjects based on age and ANA status captured in the electronic medical record have been developed and approved by the IRBs at University of Texas Southwestern Medical Center Medical Center and Medical University of South Carolina.

A limitation of this study is that one of the more interesting outcomes, prevention of evolution from ILE to SLE, is not feasible as a primary objective due to the limited study duration. Most reports suggest that 5 years would be required to see a substantial proportion of patients transition to classifiable lupus.

The SMILE trial is designed to demonstrate whether HCQ can slow the progression of disease in subjects who have ILE as measured by the accumulation of internationally defined lupus criteria. Data from this study will provide an evidence basis on which to decide whether or not HCQ should be recommended to all ILE patients, and those patients will be able to make an informed choice about the risks and benefits of this medication.

### Trial status

The current SMILE protocol version is 2.3 dated 15 October 2018. Enrollment was initiated in December 2017. Enrollment completion is anticipated for January 2020.

## Additional file

Additional file 1: SPIRIT 2013 checklist: recommended items to address in a clinical trial protocol and related documents. (DOC 137 kb)

## Abbreviations

ACR: American College of Rheumatology
AE: Adverse event
ANA: Antinuclear antibody
CAP/CLIA: College of American Pathologists/Clinical Laboratory Improvement Amendments
CLASI: Cutaneous Lupus Erythematosus Disease Area and Severity Index
CSQ: Connective Tissue Disease Screening Questionnaire
DCC: Data Coordinating Center
DSMB: Data Safety and Monitoring Board
eCRF: Electronic case report form
FDA: Food and Drug Administration
HCQ: Hydroxychloroquine
HIPAA: Health Insurance Portability and Accountability Act
IFA: Immunofluorescence assay
ILE: Incomplete lupus erythematosus
IND: Investigational new drug
NIAMS: National Institute of Arthritis and Musculoskeletal and Skin Diseases
NIH: National Institutes of Health
NSAID: Nonsteroidal anti-inflammatory drug
OMRF: Oklahoma Medical Research Foundation
PROMIS: Patient Reported Outcome Measurement Information System
RIC: Trial Innovation Network Recruitment Innovation Center
SLE: Systemic lupus erythematosus
SLEDAI: Systemic Lupus Erythematosus Disease Activity Index
SLICC: Systemic Lupus International Collaborating Clinics
UIP: University of Iowa Pharmaceuticals
